# Association between KIR-HLA combination and ulcerative colitis and Crohn’s disease in a Japanese population

**DOI:** 10.1371/journal.pone.0195778

**Published:** 2018-04-12

**Authors:** Hiromi Saito, Atsuhiro Hirayama, Takeji Umemura, Satoru Joshita, Kenji Mukawa, Tomoaki Suga, Eiji Tanaka, Masao Ota

**Affiliations:** 1 Department of Medicine, Division of Hepatology and Gastroenterology, Shinshu University School of Medicine, Matsumoto, Japan; 2 Department of Gastroenterology, Suwa Red Cross Hospital, Suwa, Japan; Duke University, UNITED STATES

## Abstract

Inflammatory bowel disease (IBD) consists of ulcerative colitis (UC) and Crohn’s disease (CD). Natural killer cell responses play a crucial role in autoimmune disease through innate immunity, in which killer cell immunoglobulin-like receptors (KIRs) are closely involved. Although the genetic combination of KIRs with their specific HLA class I ligands has been associated with IBD in Caucasians, such KIR-HLA receptor-ligand combinations are not fully understood in the Japanese. We investigated 14 KIR genes along with HLA-Bw and -C ligands in 90 patients with UC and 50 patients with CD and compared them with the characteristics of 325 healthy control subjects. The frequency of HLA-Bw4 was significantly increased in patients with UC (*P* = 1.3 × 10^−6^; odds ratio [OR] = 3.39) and CD (*P* = 0.0065; OR = 2.32) versus controls. The UC group had a significantly higher frequency of KIR2DS3 (*P* = 0.024; OR = 1.94) and lower frequency of KIR2DS4 (*P* = 0.019; OR = 0.40) and KIR2DL1-HLA-C2 (*P* = 0.035; OR = 0.47). The Tel-A/B haplotype was significantly decreased in UC patients (*P* = 0.0056; OR = 0.49). The frequency of KIR3DL1-HLA-Bw4 was significantly higher in patients with UC (*P* = 4.3 × 10^−6^; OR = 3.12) and CD (*P* = 0.0067; OR = 2.30). In conclusion, HLA-Bw4 and KIR-HLA pairs may play an important role in the genetic susceptibility to IBD in the Japanese.

## Introduction

Inflammatory bowel disease (IBD) consists of two main forms: ulcerative colitis (UC) and Crohn’s disease (CD) [1]. The pathogenesis of IBD remains elusive but is clearly influenced by both genetic and environmental factors. The dysregulation of innate and adaptive immune responses figures prominently in IBD. Natural killer (NK) cells are key components of the innate immune system primarily known for cytolytic targeting of tumor cells and virally infected cells. Killer cell immunoglobulin-like receptors (KIRs) are a family of transmembrane proteins that are expressed on NK cells and subsets of T cells. The genetic combination of KIRs with their specific HLA class I ligands regulates NK cell responses to discriminate aberrant cells from healthy ones and has been associated with autoimmune disorders, infectious diseases, and cancers. Among inhibitory KIRs, KIR2DL1 recognizes group 2 HLA-C (HLA-C2) molecules, which have lysine at position 80, KIR2DL2 and KIR2DL3 recognize group 1 HLA-C (HLA-C1) having asparagine at position 80, and KIR3DL1 recognizes HLA-Bw4 [2–6]. Although several studies have demonstrated that KIR-HLA receptor-ligand combinations are associated with IBD [7–12], discrepancies in the literature exist. KIR genes have not been evaluated in Japan to date. We therefore examined whether HLA alleles, KIR genes, and KIR-HLA combinations were associated with susceptibility to UC or CD in a Japanese population.

## Materials and methods

### Subjects

Fifty patients with CD (median age 44 years; male/female: 35/15) and 90 patients with UC (median age 47 years; male/female 50/40) were enrolled between January 2014 and August 2016. We also recruited 325 volunteer control subjects who were described previously [13]. HLA-Bw4, HLA-C1, HLA-C2, and KIR genotypes in the 325 controls were determined and reported previously [14]. The racial background of all individuals was Japanese. The diagnosis of CD [15] and UC [16] was based on existing guidelines by a combination of endoscopic, histopathological, radiological, and biochemical tests. This study was conducted according to the guidelines of the Declaration of Helsinki and was approved by the ethics committees of Shinshu University School of Medicine (No. 457) and Japan Red Cross Society Suwa Hospital (No. 26–9). Written informed consent was obtained from all subjects.

### *HLA* class I and KIR typing

Genomic DNA from patients was isolated from whole blood samples using QuickGene-800 assays (Fujifilm, Tokyo, Japan). HLA-Bw4, HLA-C1, HLA-C2, and KIR genes were typed by using PCR with sequence-specific primers [17, 18]. The KIR genotype profiles were assigned to the A/A or B/x genotypes as defined previously [19]. Genotypes for the centromeric (Cen) and telomeric (Tel) parts of the KIR locus were determined based on the presence or absence of B haplotype-defining KIR genes (i.e. A/A, A/B, or B/B) as earlier described in detail [20].

### Statistical analysis

Pearson’s chi-squared test or Fisher’s exact test were used for the analysis of categorical data. A *P* value of less than 0.05 was considered to be statistically significant after Bonferroni correction for multiple testing. Statistical analyses were performed using SPSS software version 24 (IBM, Tokyo, Japan).

## Results

The frequencies of HLA-Bw, HLA-C1, and HLA-C2 were determined in patients and adopted from our prior study in controls [14]. HLA-Bw4 was significantly more frequently found in patients with UC (72%) and CD (64%) than in controls (43%) (*P* = 1.3 × 10^−6^; OR = 3.39 and *P* = 0.0065; odd ratio [OR] = 2.32, respectively) (Table 1). HLA-Bw4 homozygosity (17% vs. 8%, *P* = 0.015; OR = 2.30) and HLA-Bw4Bw6 heterozygosity (56% vs. 35%, *P* = 0.00054; OR = 2.28) were significantly higher in patients with UC than in controls, while those with CD had a significantly higher frequency of HLA-Bw4Bw6 (52% vs. 35%, *P* = 0.024; OR = 1.98). The frequencies of HLA-C1 and -C2 were comparable among the UC, CD, and control groups.

**Table 1:** Associations of HLA-Bw4, HLA-C1, and HLA-C2 ligands with KIR in UC, CD, and control subjects.

HLA	UC (n = 90)	CD (n = 50)	Controls (n = 325)	UC vs. Controls		CD vs. Controls	
				*P*	OR (95% CI)	*P*	OR (95% CI)
Bw4	65 (72%)	32 (64%)	141 (43%)	1.3 ×10^−6^	3.39 (2.04–5.65)	0.0065	2.32 (1.25–4.30)
C1	90 (100%)	49 (98%)	320 (98%)	0.52	-	0.72	-
C2	11 (12%)	8 (16%)	68 (21%)	0.063	0.53 (0.27–1.04)	0.54	-

Data are expressed as n (%). OR: odds ratio, CI: confidence interval.

The distribution of KIR genes and their association with UC and CD patients and healthy subjects are shown in Table 2. Among the 14 KIR genes tested, the frequency of KIR2DS3 in patients with UC was higher than that in healthy controls (23% vs. 14%, *P* = 0.024; *Pc* > 0.2; OR = 1.94). Conversely, these subjects had less frequent KIR2DS4 (88% vs. 95%, *P* = 0.019; *Pc* > 0.2; OR = 0.40). No significant differences were found for KIR genes in patients with CD.

**Table 2:** Frequency of each KIR gene in 90 patients with UC, 50 patients with CD, and 325 healthy subjects.

KIR	UC n (%)	CD n (%)	Controls n (%)	UC vs. Controls	CD vs. Controls
				*P*	*P*
2DL1	90 (100%)	50 (100%)	325 (100%)	-	-
2DL2	11 (12%)	10 (20%)	47 (15%)	0.588	0.310
2DL3	88 (98%)	49 (98%)	324 (100%)	0.232	0.626
2DL4	89 (99%)	49 (98%)	324 (100%)	0.909	0.626
2DL5	34 (38%)	22 (44%)	131 (40%)	0.664	0.621
2DS1	34 (38%)	23 (46%)	129 (40%)	0.742	0.398
2DS2	12 (13%)	11 (22%)	53 (16%)	0.492	0.319
2DS3	21 (23%)	11 (22%)	44 (14%)	0.024	0.115
2DS4	79 (88%)	49 (98%)	308 (95%)	0.019	0.522
2DS5	21 (23%)	14 (28%)	93 (29%)	0.320	0.929
3DL1	83 (92%)	49 (98%)	309 (95%)	0.295	0.576
3DL2	90 (100%)	50 (100%)	325 (100%)	-	-
3DL3	90 (100%)	50 (100%)	325 (100%)	-	-
3DS1	33 (37%)	18 (36%)	136 (42%)	0.195	0.270

Data are presented as total number (%).

KIR genotype profiles were determined by the presence or absence of each KIR locus. The A/A genotype frequency did not differ among patients with UC (51%), patients with CD (46%), and healthy subjects (46%). We subdivided the KIR cluster into centromeric and telomeric regions of the A and B haplotypes (Cen-A/B and Tel-A/B) (Table 3). While there were no significant associations regarding Cen haplotypes, we found that the frequency of the Tel-A/B motif was significantly decreased in UC patients compared with controls (27% vs. 43%, *P* = 0.0056; OR = 0.49).

**Table 3:** Frequency of centromeric and telomeric KIR haplotypes in UC, CD, and control subjects.

	UC (n = 90)	CD (n = 50)	Controls (n = 325)	UC vs. Controls		CD vs. Controls
				*P*	OR (95% CI)	*P*
Centromeric						
Cen-A/A	77 (86%)	38 (76%)	272 (84%)	0.67		0.18
Cen-A/B	11 (12%)	11 (22%)	53 (16%)	0.34		0.32
Cen-B/B	2 (2%)	1 (2%)	0 (0%)	0.067		0.28
Telomeric						
Tel-A/A	55 (61%)	27 (54%)	166 (51%)	0.091	1.51 (0.93–2.42)	0.70
Tel-A/B	24 (27%)	22 (44%)	139 (43%)	0.0056	0.49 (0.29–0.82)	0.87
Tel-B/B	11 (12%)	1 (2%)	20 (6%)	0.053	2.12 (0.98–4.61)	0.39

Data are expressed as n (%). OR: odds ratio, CI: confidence interval.

We next analyzed combinations of KIRs and their HLA ligands for possible associations with susceptibility to UC or CD (Table 4). Patients with UC (69% vs. 42%, *P* = 4.3 × 10^−6^; OR = 3.12) and CD (62% vs. 42%, *P* = 0.0067; OR = 2.30) both had a significantly higher frequency of KIR3DL1-HLA-Bw4 compared with controls. Moreover, the frequency of KIR3DS1-HLA-Bw4 in UC was significantly higher than that in controls (24% vs. 15%, *P* = 0.045; OR = 1.78). In contrast, the frequency of KIR2DL1-HLA-C2 in the UC group was significantly lower than that in healthy individuals (11% vs. 21%, *P* = 0.035; OR = 0.47). Among patients with UC, the frequencies of KIR3DL1 and 2 copies of HLA-Bw4 (14% vs. 7%, *P* = 0.038; OR = 2.12) and KIR3DL1 and 1 copy of HLA-Bw4 (54% vs. 34%, *P* = 0.00047; OR = 2.30) were significantly higher than in controls. Patients with CD had a significantly higher frequency of KIR3DL1 and 1 copy of HLA-Bw4 (50% vs. 34%, *P* = 0.030; OR = 1.93) compared with healthy subjects.

**Table 4:** Frequency of KIR-HLA combinations in UC, CD, and control subjects.

KIR-HLA	UC (n = 90)	CD (n = 50)	Controls (n = 325)	UC vs. Controls		CD vs. Controls	
				*P*	OR (95% CI)	*P*	OR (95% CI)
3DL1-Bw4	62 (69%)	31 (62%)	135 (42%)	4.3×10^−6^	3.12 (1.89–5.13)	0.0067	2.30 (1.24–4.24)
3DS1-Bw4	22 (24%)	11 (22%)	50 (15%)	0.045	1.78 (1.01–3.14)	0.24	-
2DL1-C2	10 (11%)	8 (16%)	68 (21%)	0.035	0.47 (0.23–0.96)	0.42	-
2DS1-C2	5 (6%)	4 (8%)	26 (8%)	0.57	-	0.78	-
2DL2-C1	11 (12%)	10 (20%)	47 (14%)	0.59	-	0.31	-
2DL3-C1	89 (99%)	49 (98%)	324 (100%)	0.91	-	0.63	-
2DS2-C1	12 (13%)	11 (22%)	53 (16%)	0.49	-	0.32	-

## Discussion

Genome-wide association studies have revealed susceptible MHC regions [21] and several susceptibility loci outside of the MHC region for UC and CD in Japan [22–25]. The KIR locus is highly polymorphic and not well captured by genome-wide association study approaches, making KIR understudied as a susceptibility factor in autoimmune disease. The present study further examined HLA alleles as well as KIR genes in patients with IBD in a Japanese population. We detected significant associations of HLA-Bw4 alleles with UC and CD, although HLA-C1 and -C2 alleles did not differ meaningfully among UC, CD, and control groups. HLA-Bw4Bw6 was significantly associated with both UC and CD, with a significantly higher frequency of HLA-Bw4 homozygosity for UC. Since no significant associations of HLA-Bw4, -C1, or -C2 with IBD have been reported in Caucasians, HLA-Bw4 might be an important susceptibility allele for IBD in Japanese patients.

Our data showed that KIR2DS3 increased and KIR2DS4 decreased the risk of developing UC, in contrast to increased frequencies of KIR2DL2 and KIR2DS2 in Caucasian UC [7]. Moreover, the frequency of the Tel-A/B haplotype in UC patients was lower than that in controls, which suggested that KIR haplotype was responsible for UC resistance in our cohort. Conversely, Tel-haplotypes were not significantly associated with CD. Diaz-Pena et al. [11] examined KIR haplotypes in Spanish patients with CD and found significant associations for the A/A genotype and Cen-A/A. Such discrepancies may stem from differences in ethnicities and sample number.

The most significant finding in this study was the association between KIR-HLA receptor-ligand pairings and IBD development. Specifically, the KIR3DL1-HLA-Bw4 combination was significantly associated with UC and CD in our cohort. In our previous studies, KIR3DL1-HLA-Bw4 was also related to a response to interferon-based antiviral therapy in the Japanese [20, 26]. Yawata et al. reported that KIR3DL1 polymorphisms modulated a major NK cell effector function to demonstrate their functional importance and revealed the effects of cognate 3DL1-Bw4 interactions to be modulated by KIR3DL1 polymorphisms [27]. As we did not address KIR allotypes, further studies are required.

Moreover, our results uncovered a protective role for KIR2DL1-HLA-C2 against UC in a Japanese series. Although KIR2DL2-HLA-C1 and KIR2DL2/2DL3-HLA-C1C1 have been associated with IBD in Caucasians [7–9, 11], our findings showed no associations of these KIR-HLA pairs with IBD. Regarding KIR inhibitory combinations, KIR2DL1-HLA-C2 interactions are considered the strongest, followed next by KIR2DL2-HLA-C1 and KIR2DL3-HLA-C1 [28]. Functional studies on NK cells are needed to assess their precise mechanisms.

This investigation had several limitations. It was a single-center study with a relatively small sample size and smaller female ratio of CD patients than in the normal population. To the best of our knowledge, however, it is the first addressing associations of KIR-HLA pairs with IBD in Japan.

In summary, the present study revealed HLA-Bw and KIR-3DL1-HLA-Bw4 associations with susceptibility to UC and CD in a Japanese population. Accordingly, KIR-HLA pairs may play an important role in the pathogenesis of Japanese IBD and further studies are warranted to clarify their involvement in UC and CD.
